# Inhibition of JAK-STAT Signaling with Baricitinib Reduces Inflammation and Improves Cellular Homeostasis in Progeria Cells

**DOI:** 10.3390/cells8101276

**Published:** 2019-10-18

**Authors:** Chang Liu, Rouven Arnold, Gonçalo Henriques, Karima Djabali

**Affiliations:** Epigenetics of Aging, Department of Dermatology and Allergy, TUM school of Medicine, Technical University of Munich (TUM), 85748 Garching, Germany; c.liu@tum.de (C.L.); rouven.arnold@tum.de (R.A.); goncalohenriques26@gmail.com (G.H.)

**Keywords:** Progerin, Baricitinib, Senescence, JAK-STAT, inflammation, age-related disease, Progeria

## Abstract

Hutchinson-Gilford progeria syndrome (HGPS), a rare premature aging disorder that leads to death at an average age of 14.7 years due to myocardial infarction or stroke, is caused by mutations in the *LMNA* gene. Nearly 90% of HGPS cases carry the G608G mutation within exon 11 that generates a truncated prelamin A protein “progerin”. Progerin accumulates in HGPS cells and induces premature senescence at the cellular and organismal levels. Children suffering from HGPS develop numerous clinical features that overlap with normal aging, including atherosclerosis, arthritis, hair loss and lipodystrophy. To determine whether an aberrant signaling pathway might underlie the development of these four diseases (atherosclerosis, arthritis, hair loss and lipodystrophy), we performed a text mining analysis of scientific literature and databases. We found a total of 17 genes associated with all four pathologies, 14 of which were linked to the JAK1/2-STAT1/3 signaling pathway. We report that the inhibition of the JAK-STAT pathway with baricitinib, a Food and Drug Administration-approved JAK1/2 inhibitor, restored cellular homeostasis, delayed senescence and decreased proinflammatory markers in HGPS cells. Our ex vivo data using human cell models indicate that the overactivation of JAK-STAT signaling mediates premature senescence and that the inhibition of this pathway could show promise for the treatment of HGPS and age-related pathologies.

## 1. Introduction

Hutchinson-Gilford progeria syndrome (HGPS, OMIM 176670) is a rare fatal pediatric disorder characterized by segmental severe premature aging and early death for which no cure exists [1]. Patients suffering from HGPS show clinical features resembling physiological aging, including vascular disease, lipodystrophy, alopecia, progressive joint contractures and stiffness, and osteoporosis, that lead to shortened life span and death at approximately 14.7 years of age [1,2]. The genetic basis of HGPS is linked to mutations in the *LMNA* gene [3]. In the majority of HGPS cases, a single de novo mutation (LMNA 1824C >T, G608G) activates a cryptic splicing site, causing the production of a truncated prelamin A protein with a 50 amino acid deletion called progerin. Progerin lacks the cleavage site for zinc-metalloproteinase (ZMPSTE24) and therefore remains farnesylated, causing altered gene expression, DNA damage, mitochondrial dysfunction, defective proteostasis and oxidative stress which cause cells to enter premature senescence [4]. 

Among all of the traits that characterize HGPS patients, we focused on the four conditions typically recognized, namely, vascular disease, arthritis, lipodystrophy, and alopecia. These pathologies are not specific to HGPS, as these conditions also develop in patients suffering from other progeroid syndromes, such as in cases of mandibuloacral dysplasia (MAD), restrictive dermopathy (RD), and Malouf syndrome [5,6]. To examine whether these four conditions might share common defective molecular mechanisms, we investigated the literature to find the occurrence of these pathologies in different combinations in individuals other than HGPS patients. Indeed, the incidence of these four pathologies is not restricted to HGPS; for instance, vascular disease and alopecia are observed in patients with severe androgenetic alopecia (AGA) [7] or cerebral autosomal recessive arteriopathy with subcortical infarct and leukoencephalopathy (CARASIL) [8]. Atherosclerosis and loss of subcutaneous fat occur in congenital generalized lipodystrophy and in patients with HIV-associated lipodystrophy syndrome [9,10]. Rheumatoid arthritis and alopecia or lipodystrophy are observed in patients with juvenile dermatomyositis [11]. Hence, these four conditions also affect normal elderly individuals albeit rarely all together.

The cooccurrence of these four age-related diseases prompted us to investigate whether these pathologies could result from a shared imbalanced signaling pathway or converging pathways. Several studies on HGPS have reported alterations in different signaling pathways, including the mammalian target of rapamycin (mTOR) [12], retinoblastoma protein (pRb) [13], nuclear factor kappa B (NF-κB) [14] and nuclear factor erythroid 2–related factor 2 (Nrf2) [15,16]. However, how these pathways initiate the development of HGPS and particularly these four pathologies remains unknown. 

To gain additional insight into HGPS pathogenesis, we tested our hypothesis that converging signaling pathway(s) might underlie the development of the four conditions, namely, vascular disease, arthritis, lipodystrophy, and alopecia by performing a text mining analysis of scientific literature and databases to identify genes reported to be altered in each of these four distinct pathologies. This text mining approach identified a unique set of 17 genes that were found to be altered in all four pathologies. Analyses of the 17 genes using bioinformatics showed that all 17 entities were interconnected and therefore belonged to converging signaling pathways. Furthermore, 14, out of these 17 genes encoded for proinflammatory factors that are known targets of Janus kinase (JAK)–signal transducer and activator of transcription (STAT) signaling. Using an ex vivo cell-based aging model, we demonstrated that the 17 genes, including the 14 genes encoding proinflammatory factors and targets of JAK-STAT signaling, were altered in HGPS and in normal cells during replicative senescence and during DNA damage induced senescence. Our study indicates that the JAK1/2-STAT1/3 pathway is overactivated in premature cellular aging. Moreover, we show that the inhibition of JAK-STAT signaling with baricitinib (Bar) a Food and Drug Administration (FDA)-approved JAK1/2 inhibitor [17], significantly decreased proinflammatory factors, delayed senescence and rebalanced cell homeostasis in senescing HGPS cells. 

## 2. Materials and Methods

### 2.1. Text Mining Study

A straightforward data mining procedure to identify candidate genes related to one of the four diseases regarded as the main phenotypes of HGPS was used. A keyword search was performed using PubMed (http://www.pubmed.gov) as the main source. To automate the task of searching for all possible connections between each of the four diseases among all genes in the human genome from HGNC [18] (latest version of 01.01.2019 listed 19,194 protein-coding genes), a search algorithm was developed by using the programming language R. The basic structure was created with two R packages. The “RISmed” package (https://cran.r-project.org/web/packages/RISmed/index.html) allowed us to extract bibliographic content from the National Center for Biotechnology Information (NCBI) database. We also applied the “pubmed.mineR” package (https://cran.r-project.org/web/packages/pubmed.mineR/) to text mine PubMed abstracts [19,20]. This pooled all papers associated with one of the four diseases and its link to a particular gene. Since the cooccurrence of a gene and a disease in an abstract does not necessarily provide evidence, the data were checked manually to determine whether the papers truly showed a regulatory link. 

### 2.2. Identification of the Signaling Pathways

**Figure 1:**
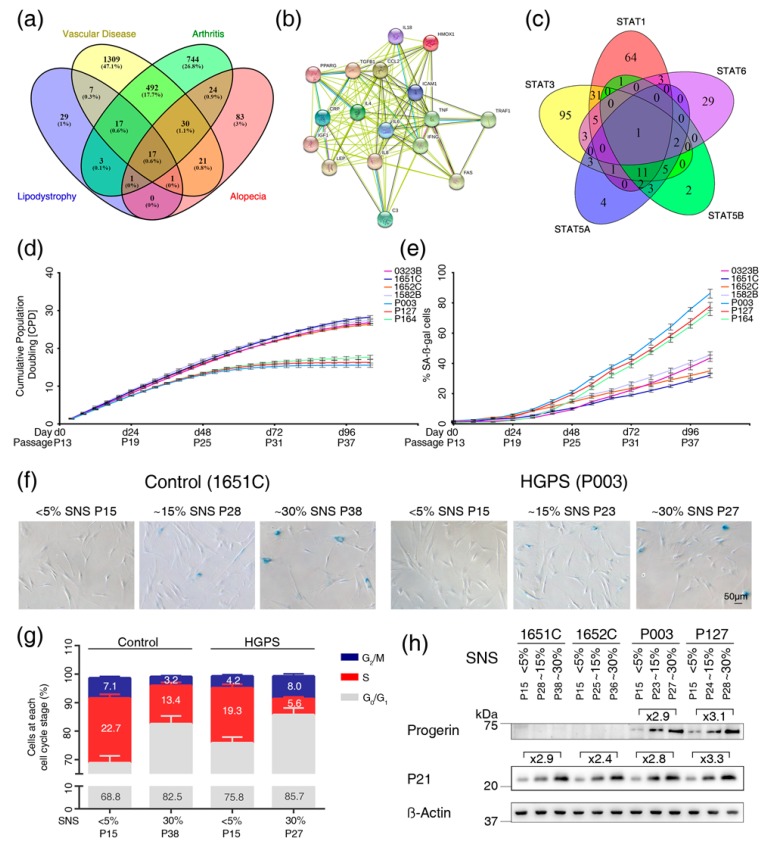
Text mining analysis to identify genes associated with vascular disease, arthritis, alopecia and lipodystrophy, and characterization of the cell-based aging model. (**a**) Venn diagram of the 2778 genes linked to the four diseases as determined by text mining. (**b**) Interaction network of the 17 gene products visualized by STRING. Nodes are proteins, and lines represent functional associations between proteins. A green line indicates neighborhood evidence; a blue line indicates cooccurrence evidence; a purple line indicates experimental evidence; a light blue line indicates database evidence; a black line indicates coexpression evidence. (**c**) Venn diagram of the signal transducer and activator of transcription (STAT) gene family showing the number of genes that are regulated by different STATs as indicated by the corresponding circles. Each gene was searched in the TRRUST version 2 (https://www.grnpedia.org/trrust/), TfactS (http://www.tfacts.org/) and Regulatory Circuits databases (http://regulatorycircuits.org/). Altogether, a total of 269 genes were found and curated to prevent repetitions due to aliases. (**d**) Growth curves of four independent control cell strains (purple, blue, orange, gray) and three Hutchinson-Gilford progeria syndrome (HGPS) cell strains (light blue, red, and turquoise). All cultures started at passage 13, and at this passage, the percentage of SA-ß-gal-positive cells was less than 5% in all cell strains. Proliferation rates were determined over 26 passages over 104 days. (**e**) The percentage of SA-ß-gal-positive cells was scored every other passage in cultures from panel d. (**f**) Representative images of SA-ß-gal-positive cells in cultures exhibiting <5% SNS, 15% SNS and 30% SNS. GMO1651c corresponds to normal fibroblasts and P003 corresponds to HGADFN003, an HGPS cell strain. (**g**) Relative percentage of cells in the G_0_/G_1_, S and G_2_/M phases of the cell cycle are shown for normal (1651c) and HGPS (P003) cultures with a senescence index of <5% and 30%. PI was used for DNA staining. (**h**) Representative western blot of control (1651c, 1652c) and HGPS (P003, P127) fibroblasts from cultures at the indicated percentages of senescence. Antibodies directed against the indicated proteins were used. All experiments were performed at least three times (*n* > 3).

All genes showing a connection to one of the four diseases were analyzed in a Venn diagram to determine the list of genes associated with all four diseases (Figure 1a). In total, 17 genes were associated with the four diseases. To show how these genes were functionally interconnected, a protein-protein interaction network was obtained using STRING analysis (https://string-db.org/).

Next, to determine whether these genes occur in the same signaling pathways, we searched for the regulators and targets of the corresponding proteins using the following databases: (1) TTRUST version 2 (http://www.grnpedia.org/trrust/), (2) TfactS (www.tfacts.org/), and (3) Regulatory Circuits (http://regulatorycircuits.org/).

All data and references were also manually curated. The lists of genes regulated by the STAT family were established using the abovementioned databases. Aliases and repetitions were removed by using Excel (Microsoft, Redmond, WA, USA).

### 2.3. Cell Culture and Drug Treatments

All fibroblasts from patients with HGPS were obtained from The Progeria Research Foundation Cell and Tissue Bank (http://www.progeriaresearch.org). The following fibroblasts were used: HGADFN003 (2-year-old male), HGADFN127 (3-year-old female), HGADFN164 (4-year-old female), and HGADFN188 (2-year-old-female). Control fibroblasts were obtained from the Coriell Institute for Medical Research (Camden, NJ, USA). The following cell strains were used: GM01651C (13-year-old female), GM01652C (11-year-old-female), GM01582B (11-year-old female), GM00323B (11-year-old male), and GM03349C (10-year-old male). Cells were grown in Dulbecco’s modified Eagle’s medium + GlutaMAX™ (DMEM, Gibco™, Thermo Fisher Scientific Inc., Waltham, MA, USA), supplemented with 15% fetal bovine serum (FBS, Gibco™, Thermo Fisher Scientific Inc., Waltham, MA, USA) 1% L-glutamine (Gibco™, Thermo Fisher Scientific Inc., Waltham, MA, USA), 1% penicillin-streptomycin (10,000 U/mL, Gibco™, Thermo Fisher Scientific Inc., Waltham, MA, USA), and 0.5% gentamicin (10 mg/mL, Gibco™, Thermo Fisher Scientific Inc., Waltham, MA, USA).

Baricitinib (Absource Diagnostics GmbH, Munich, Germany) was added to the culture media at a concentration of 1 μM every 2–3 days for the indicated period. Mock-treated fibroblasts were cultured in parallel with media containing the vehicle (DMSO). 

For etoposide treatment, cells were pretreated with or without 1 µM of Bar for two days and then changed to medium with or without etoposide in the presence or absence of Bar for a period of six days. Next, the cells were grown for four days with or without Bar as indicated.

### 2.4. Determination of Cumulative Population Doubling Determination

Cells were seeded at a density of 1.5 × 10^5^ cells per 10-cm dish and counted every four days with a Muse™ Cell Analyzer (Merck Millipore, Burlington, MA, USA) using DNA binding dyes to assess the fluorescent signal. Cumulative population doublings (CPDs) were determined using the following formula:

(1) n=3.32 (logcells harvested−logcells seeded)+x,

where *n* = the final CPD number at the end of a given subculture, and *x* = the former CPD as described previously [15].

### 2.5. Senescence Associated Beta-Galactosidase Assay

Senescence was assessed in HGPS and control cultures at each passage using a SA-β-Gal senescence staining kit (CS0030-1KT, Sigma-Aldrich, St. Louis, MO, USA) according to the manufacturer’s instructions. Blue cells were counted using the Cell Counter plugin from ImageJ. A total of 1000 cells were counted from each sample at each passage.

### 2.6. Cell Cytotoxicity

A CellTox™ Green Cytotoxicity Assay kit (Promega, Madison, WI, USA) was used to assess the toxicity of Bar by measuring the changes in membrane integrity that occur as a result of cell death using an asymmetric cyanine dye. A concentration of 1 μM Bar was selected for all experiments. 

### 2.7. Cell Cycle Analysis

A Muse Cell Cycle Assay kit (MerckMillipore, Burlington, MA, USA) was used to quantify the percentage of cells in the G_0_/G_1_, S and G_2_/M phases of the cell cycle. The kit contains a premixed reagent, which includes the nuclear DNA intercalating stain propidium iodide (PI) and RNAse A. By staining DNA content, cells at different stages of the cell cycle can be identified. The signal was quantified using a Muse Cell Cycle Analyzer (MerckMillipore, Burlington, MA, USA).

### 2.8. Measurement of Proteasome Activity

To detect proteasome activity in cell lysates a 20S Proteasome Assay kit (Cayman Chemical Company, Ann Arbor, MI, USA) was used. An equal number of cells (32,000) was used for each sample. The assay was performed according to the manufacturer’s instructions. Briefly, the kit employs a specific 20S substrate that generates a highly fluorescent product upon cleavage by the active enzyme. The signal was measured at an excitation wavelength of 485 nm and an emission wavelength of 520 nm. All measurements were made in triplicate in at least three independent experiments.

### 2.9. Autophagy Measurement

Autophagy was measured using Cayman’s Autophagy/Cytotoxicity Dual Staining Kit (Cayman Chemical Company, Ann Arbor, MI, USA) employing monodansylcadaverine (MDC), an autofluorescent substance used for the detection of autophagic vacuoles in cultured cells. Equal numbers of cells (32,000) were seeded in quintets in 96-well plates. After cells attached during an overnight incubation (37 °C), MDC was added to the wells at a 1:1000 ratio. Measurements of the autophagic vacuole intensities were obtained using an excitation wavelength of 355 nm and an emission wavelength of 520 nm. All measurements were repeated in at least three independent experiments.

### 2.10. Measurement of ROS

To measure ROS, we used the Cellular ROS Detection Assay Kit (Abcam, Cambridge, UK). The kit includes a fluorescent dye as an oxidative stress detection reagent, which is designed to directly monitor real-time ROS. Equal numbers of cells (32,000) were seeded in quintets in 96-well plates. After attachment, the cells were incubated with 25 μM 2′,7′-dichlorofluorescein diacetate (DCFDA) for 45 min at 37 °C. The fluorescence was then measured at the excitation and emission wavelengths of 485 nm and 520 nm respectively after a washing step. All measurements were repeated in at least three independent experiments.

### 2.11. Measurement of Intracellular ATP

ATP quantification was performed using a CellTiter-Glo^®^ Luminescent Cell Viability Assay Kit (Promega, Madison, WI, USA). As an indicator of metabolically active cells, ATP generates a luminescent signal in this assay. Equal numbers of cells (32,000) were seeded in quintets in 96-well plates. After 12 h, the cells were incubated with 100 μL CellTiter-Glo reagent for 10 min, and luminescence intensity was measured. An ATP standard was assessed in parallel. All measurements were repeated in at least three independent experiments.

### 2.12. Gene Expression Analysis

RNA extraction from cell pellets was performed using the GeneJET RNA Purification Kit (Thermo Fisher Scientific Inc., Waltham, MA, USA). Approximately 1 × 10^6^ dermal fibroblasts were collected for RNA preparation. RNA quantity and purity were assessed using a Nanodrop spectrophotometer (NanoDrop ND-1000, Thermo Fisher Scientific Inc., Waltham, MA, USA). In total, 1000 ng of RNA was reverse-transcribed into cDNA, using a High-Capacity cDNA Reverse Transcription Kit (Thermo Fisher Scientific Inc., Waltham, MA, USA). Real-time PCR primers were designed by using NCBI/Primer-BLAST [21]. All primers were purchased from ThermoFisher. All evaluated genes and their corresponding primers are shown in the primer list (Table S4). Real-time PCR was performed using the PowerUpTM SYBR^TM^ Green Master Mix (Applied Biosystems^TM^, Thermo Fisher Scientific Inc., Waltham, MA, USA) in a StepOnePlus^TM^ Real-Time PCR System Thermo Fisher Scientific Inc., Waltham, MA, USA). For optimal detection, we used 300 nM of each primer and 50 ng of the template in a 20-μL reaction volume. The thermal profile consisted of an initial denaturation step at 95 °C for 20 s followed by 45 cycles of 95 °C for 3 s and 60 °C for 30 s. Amplification signals were all observed between cycles 10 and 40. All experiments were performed in at least three replicates and at least three independent experiments. GAPDH was used as an endogenous control.

### 2.13. Western Blots Analysis

Adherent cells were harvested by scrapping. A Bradford assay was performed to estimate the protein concentration. A total of 25 µg of protein in Laemmli sample buffer (BioRad, Hercules, CA, USA) was resolved on precast protein gels (4–20% Mini-PROTEAN^®^ TGX^TM^) and transferred onto nitrocellulose membranes. After blocking in 5% nonfat milk, the membranes were incubated overnight at 4 °C with the following primary antibodies: anti-JAK1 (cat. no. 3332, Cell Signaling, Danvers, MA, USA, 1/1000), anti-JAK2 (cat. no. 3230, Cell Signaling, 1/1000), anti-STAT1 (cat. no. 14994, Cell Signaling, 1/1000), anti-P-STAT1 (cat. no. 9167, Cell Signaling, 1/1000), anti-STAT3 (cat. no. 9139, Cell Signaling, 1/1000), anti-P-STAT3 (cat. no. 9145, Cell Signaling, 1/1000), anti-P21 (cat. no. MA5-14949, Invitrogen, Carlsbad, CA, USA, 1/1000), anti-ß-actin (Sigma-Aldrich, 1/5000), anti-progerin (rabbit monoclonal Ab, 0.1 µg/mL) [13], and anti-lamin A/C (cat. no. sc-20681, Santa Cruz Biotechnology, Dallas, TX, USA, 1/10,000). After washing in TBS Tween, the membranes were incubated with a corresponding secondary antibody conjugated with horseradish peroxidase (Jackson ImmunoResearch Laboratories, West Grove, PA, USA). For signal amplification, we used the enhanced chemiluminescence detection system (ECL substrate; BioRad). Staining was visualized using a ChemiDoc™ MP, and densitometry was performed using ImageJ software (NIH). Protein signals were quantified by normalizing to ß-actin as indicated. A mild stripping buffer was used to reuse the membranes.

### 2.14. Immunocytochemistry

For immunocytochemistry, fibroblasts were grown on coverslips, fixed with 100% methanol at −20 °C for 10 min and were further processed for immunohistochemistry as previously described reports [15]. The following primary antibodies were used: anti-progerin (1 μg/mL [15], anti-lamin A/C (cat. no. MA3-1000, Invitrogen, Carlsbad, CA, USA, 1/100). The secondary antibodies used were affinity-purified Alexa Fluor 488 donkey IgG antibodies (Molecular Probes, Eugene, OR, USA) and Cy3-conjugated IgG antibodies (Jackson ImmunoResearch). DAPI in Vectashield mounting medium (Vector Inc., Tokyo, Japan) was used for counterstaining. The images were acquired (exposure time-matched) using an Axioplan fluorescence microscope (Carl Zeiss, Oberkochen, Germany).

### 2.15. Statistics

Comparative analysis of the different characteristics of HGPS cells and healthy controls (treated and untreated) was conducted using Student’s t test (*n* ≥ 3, * *p* < 0.05, ** *p* < 0.01, *** *p* < 0.001). *p* < 0.05 was considered to be statistically significant. Data were expressed as the mean ± standard deviation (mean ± SD). Prism version 6.01 (GraphPad, San Diego, CA, USA) was used to perform calculations and to create graphs. mRNA contents were calculated by pairwise fixed reallocation randomization test [22].

## 3. Results

### 3.1. Text Mining Analysis to Identify Genes Altered in Vascular Disease, Arthritis, Alopecia and Lipodystrophy

The four diseases, alopecia, lipodystrophy, arthritis and vascular disease, are commonly recognized as the main trait characteristics of HGPS. Therefore, we set up a data mining method to identify genes involved in all four of these conditions. We conducted text mining using keyword searches in the scientific literature in PubMed (http://www.pubmed.gov). We searched for all possible variations of each of the four diseases and all genes in the human genome from the Human Gene Nomenclature Committee (HGNC) [18] as described in the Materials and Methods.

The text mining search identified 2843 genes associated with at least one of the four-targeted diseases. In total 157 genes were associated with alopecia, 67 genes were associated with lipodystrophy, 1049 genes were associated with arthritis, and 1560 genes were associated with vascular disease (Table S1). We generated a Venn diagram to identify genes that were altered in all of the four diseases (Figure 1a). Seventeen genes (complement 3 (C3), chemokine (C-C motif) ligand 2 (CCL2/MPC1), C-reactive protein (CRP), C-X-C motif chemokine ligand 8 (CXCL8/IL8), cell surface death receptor (FAS), heme oxygenase 1 (HMOX1), intercellular adhesion molecule 1 (ICAM1), insulin-like growth factor 1 (IGF1), interleukin 18 (IL18), interleukin 4 (IL4), interleukin 6 (IL6), interferon gamma (IFNG), peroxisome proliferator activated receptor gamma (PPARG), transforming growth factor beta 1 (TGFB1), tumor necrosis factor alpha (TNFα), leptin (LEP) and TNF receptor-associated factor 1 (TRAF1)) showed an association with all four conditions (Figure 1a). This finding suggested that these 17 genes might be deregulated in HGPS.

To determine the expression pattern of the 17 genes in each of the four diseases, we further curated the text mining-derived publications. Table S2 summarizes the function and predicted expression pattern of each of the 17 genes in relation to the four diseases.

PPARG, IGF1, and HMOX1 were downregulated, whereas CCL2, CXCL8, ICAM1, CRP, TRAF1, IL18, IL6, TNFα, TGFB1 and FAS were upregulated in the four conditions (Table S2). Most of these 17 genes encode known cytokines, including the proinflammatory components CRP, IL6, CXL8/IL8, CCL2/MCP1, and TNFα, factors classified as senescence-associated secretory phenotypes (SASPs) that are increased in most age-related diseases [23,24]. Therefore, the text mining results suggest that these four conditions might share a common molecular mechanism: the presence of chronic inflammation. In support of this assumption, STRING analysis, a protein-protein interaction network builder (https://string-db.org), showed that all 17 proteins were functionally connected and could play a role in the development of these four pathologies (Figure 1b).

To identify the signaling pathway(s) that modulate these 17 genes, we searched for regulators and targets of these 17 entities using three databases: TRRUST version 2 [25], TfactS [26] and Regulatory Circuits [27]. These analyses identified the transcription factors nuclear factor kappa B subunit 1 (NF-kB1), Rela proto-oncogene (Rela), STAT1, STAT3, STAT5a, STAT5b and STAT6 as part of the regulatory network that could modulate several of these genes (Table 1). These results support the involvement of the NF-kB signaling pathway via NFkB1 and Rela, and the involvement of the JAK-STAT signaling pathway via STAT1, STAT3, STAT5a, STAT5b, and STAT6 in the development of the four pathologies. The link to the NF-kB pathway was not surprising because previous studies have demonstrated that the activation of NF-kB pathway promotes aging and age-related conditions [28]. However, the link to STAT transcription factors was novel and suggested that alterations in the JAK-STAT pathway could mediate these four pathologies including HGPS.

To determine which STATs regulate the 17 identified genes, we established lists of genes regulated by each of the STAT proteins using the TRRUST version 2, TfactS and Regulatory Circuits databases [25,26] (Table S3). We identified 269 genes as targets of the STAT family of proteins, 119 of which were regulated by STAT1, 10 by STAT2, 157 by STAT3, 14 by STAT4, 32 by STAT5a, 27 by STAT5b and 43 by STAT6 (Table S2). Using a Venn diagram, we determined the list of genes that could be regulated by multiple STATs as indicated by overlapping circles (Figure 1c). Fourteen of the 17 genes were targets of STAT1 and STAT3 (Figure 1c and Table 1).

Collectively, the results of the text mining study indicate that increased proinflammatory factors are a potential common etiology among the four distinct diseases. These factors were linked to JAK-STAT signaling, suggesting that the overactivation of JAK-STAT signaling might induce the development of these four diseases.

### 3.2. Cell-Based Aging Model to Investigate Normal and Premature Cellular Aging

To establish our ex vivo aging model to compare replicative senescence in HGPS cells to that in normal, we first compared HGPS primary fibroblast cultures at different passages with control cultures from matching passage numbers to track cellular lifespan in vitro. However, this comparison was not reliable because HGPS cells exhibit a decreased growth rate earlier than do normal cells (Figure 1d), as previously reported [29]. In culture, the loss of proliferative potential is asynchronous; there can be growth-arrested cells in the early passages and dividing cells in the later passages [30]. To standardize our long-term replicative senescence cultures, we scored the growth rate at each passage and determined the percentage of senescent cells (SNS) every other passage (Figure 1d,e). The percentage of SNS was determined using the senescence-associated beta-galactosidase (SA-β-Gal) assay (Figure 1e,f) as previously described [31]. All primary fibroblast strains used in this study were initiated at P13 and exhibited ≤ 5% SNS. 

**Figure 2:**
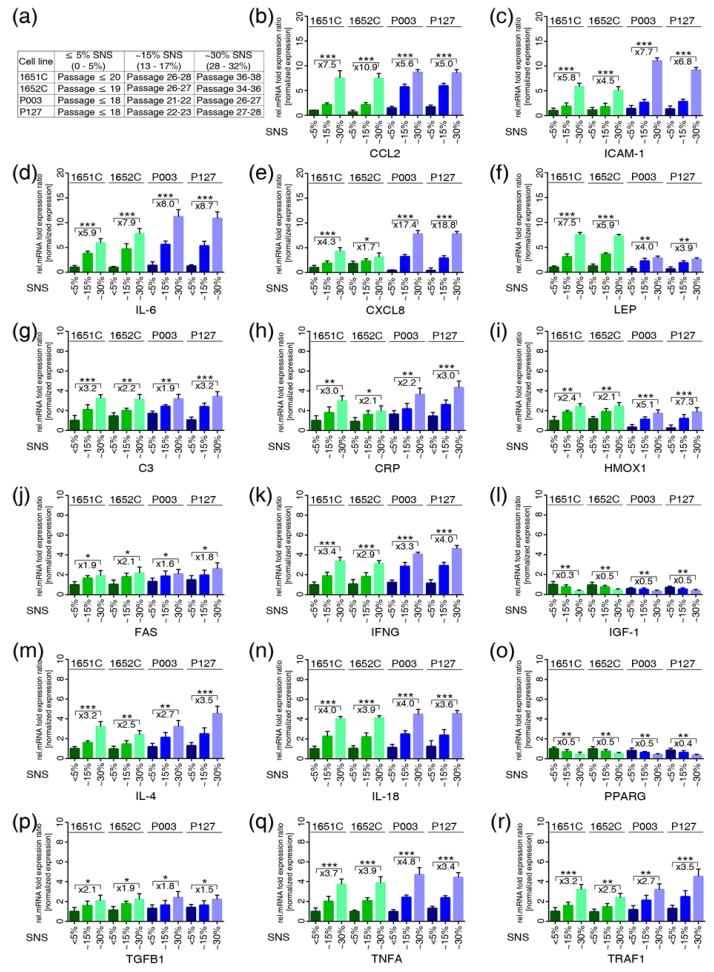
Quantitative real-time PCR analysis of the 17 genes identified by text mining in normal and HGPS cells during replicative senescence. (**a**) Table showing the cell strains and the passages corresponding percentage of senescence (SNS). (**b**–**r**) mRNA levels of the indicated genes were determined in controls (GMO1651c, and GMO1652c) and HGPS (HGADFN003 and HGADFN127) cell strains at the indicated percentage of senescence (SNS). Relative expression was normalized to the expression of GAPDH. Graphs show the mean ± SD (* *p* < 0.05, ** *p* < 0.01, *** *p* < 0.001, *n* > 3). The mean fold changes between 5% and 30% SNS for control and HGPS are indicated.

The growth rates started to decrease in HGPS cultures after six to seven passages corresponding to P19-P20 at which time these cultures exhibited 5% to 8% SNS (Figure 2d,e). In contrast, normal cells at matching passages (P19-P20) continued to grow exponentially (Figure 1d,e). Scoring the percentage of SNS every other passage over long-term culture demonstrated that at matching passage numbers, the percentage of SNS was higher in HGPS cultures than in normal cultures. Therefore, the senescence index of the cultures is used in further studies as a critical parameter to evaluate changes relative to HGPS cells by comparison to normal cells.

To further support the use of the senescence index of the cultures, we monitored the cell cycle of HGPS and normal fibroblast cultures exhibiting ≤ 5% SNS and ~30% SNS (Figure 1g). Analyses of cultures with ≤ 5% SNS confirmed that the growth of HGPS cells is slower than that of control cells, as indicated by the reduced number of HGPS cells in S phase (Figure 1g). In cultures with ~30% SNS, the population of cells in G0/G1 phase was similar in control and HGPS cultures (Figure 1g). Moreover, western blot analyses confirmed that HGPS and control cultures with similar SNS indexes exhibited comparable levels of p21, another marker of senescence (Figure 1h) [32]. Because HGPS cells express progerin, which accumulates and disrupts the nuclear architecture [33], we probed progerin during replicative senescence (Figure 1h). While progerin was not detected in normal cells, it accumulated in senescing HGPS cells as previously suggested [33].

### 3.3. Profiles of the 17 Genes Associated with Vascular Disease, Arthritis, Lipodystrophy and Alopecia in the Cell-Based Aging Model

We determined the gene expression profiles of the 17 genes (Figure 2) using the ex vivo cell-based aging model by monitoring the senescence index of the cultures (Figure 2a). The direction of changes and the fold changes in the expression levels of C3, CRP, FAS, IFNG, IGF1, IL6, IL4, IL18, PPARG, TGFB1, TNFα, and TRAF1 were remarkably similar in both normal and HGPS cultures with similar senescence indexes (Figure 2). Increased CCL2/MCP1 mRNA levels were detected at an earlier time point in HGPS cultures (15% SNS) than in normal cultures (~30% SNS) (Figure 2b). The levels of ICAM1 and CXCL8/ IL8 were higher in HGPS cultures with ~30% SNS (Figure 2c,e). However, LEP was markedly increased in normal cultures (~30% SNS, 7.20-fold, p=0.002) compared with HGPS (~30% SNS, 3.42-fold, p=0.013) cultures (Figure 2f). In accordance with the text mining results, IGF-1 and PPARG were decreased in the cultures of both cell types at ~15% and ~30% SNS (Figure 2i,o).

Taken together, these findings indicate that the regulation of the 17 genes during replicative senescence in both normal and HGPS cells was consistent with the predicted text mining results (Table S2). Moreover, both normal and HGPS cells exhibited a similar proinflammatory secretory phenotype during replicative senescence, although HGPS cells entered senescence earlier than did normal cells.

### 3.4. Overactivation of JAK-STAT Signaling During Replicative Senescence in Normal and HGPS Fibroblasts

Prompted by the increased expression of inflammatory cytokines during cellular aging, we investigated the status of JAK-STAT signaling, a key regulator of cytokine production [34]. The mRNA expression profiles indicated that the mRNA levels of JAK1, JAK 2, STAT1 and STAT3 were increased in both normal and HGPS cells from cultures with ~30% SNS (Figure S1a,b). However, JAK3, TYK2 and the other STATs showed no obvious changes in either cell type (Figure S1a,b). These findings indicate a potential role for JAK1/2-STAT1/3 signaling in the cellular aging of normal and HGPS cells.

**Figure 3:**
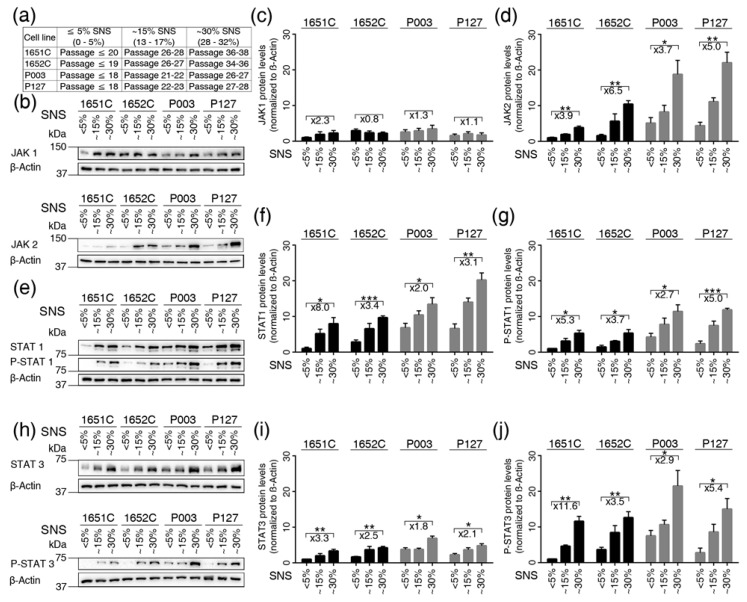
Status of the JAK-STAT signaling pathway in control and HGPS cells during replicative senescence. (**a**) Table showing the cell strains and the passages corresponding percentages of senescence (SNS). (**b**,**e**,**h**) Representative western blot images for JAK1/2, STAT1/3, p-STAT1 (tyr701), p-STAT3 (tyr705) and β-actin in the control (GMO1651c, and GMO1652c) and HGPS (HGADFN003, and HGADFN127) cell strains at the indicated percentages of senescence. (**c**) Quantification of JAK1, (**d**) JAK2, (**f**) STAT1, (**g**) p-STAT1, (**i**) STAT3, and (**j**) p-STAT3. Graphs show the mean ± SD. (* *p* < 0.05, ** *p* < 0.01, *** *p* < 0.001, *n* > 3). Fold changes between the samples with 5% and 30% senescence are indicated.

Next, we evaluated the levels of JAK1, JAK2, STAT1 and STAT3 proteins by western blot analyses (Figure 3). The levels of JAK1 in young (≤ 5% SNS) cultures varied between the different cell strains and showed no significant changes during replicative senescence (~15%, ~30% SNS) (Figure 3b,c). In contrast, JAK2 protein levels increased in a cellular-age-dependent manner in both control and HGPS cells (Figure 3b,d). Then, we examined the status of STAT1 and STAT3 during replicative senescence (Figure 3e–j). STAT1 protein levels were higher than STAT3 levels; however, the levels of both proteins increased over time in normal and HGPS cells, as observed in cultures with ~30% SNS (Figure 3f,i). Upon JAK activation, STAT1 becomes phosphorylated on tyrosine residue 701, and STAT3 is phosphorylated on tyrosine 705. Western blots indicated that both STAT1 and STAT3 were phosphorylated on these tyrosine residues during replicative senescence in both cell types (Figure 3e,h). Hence, their phosphorylation levels increased as the senescence index increased in both normal and HGPS cultures (Figure 3e–j). Collectively, these data indicate the overactivation of JAK-STAT signaling during cellular aging in HGPS and normal cells. 

### 3.5. Baricitinib, a Specific Inhibitor of JAK1 and JAK2, Efficiently Blunts STAT1 and STAT3 Activation

To block the JAK1/2-STAT1/3 pathway, we used Bar (LY3009104), a small molecule that selectively and reversibly inhibits the JAK1 and JAK2 enzymes [17]. We first assessed the cytotoxicity of Bar and selected at the dose of 1 μM, which showed similar basal toxicity to mock treatment in normal cells (Figure S2a). Hence, treatment of normal and HGPS cultures with 1 μM Bar for 96 h without changing the medium indicated that Bar was stable and effectively inhibited JAK1/2 (Figure S2b–f), as previously reported [17]. 

**Figure 4:**
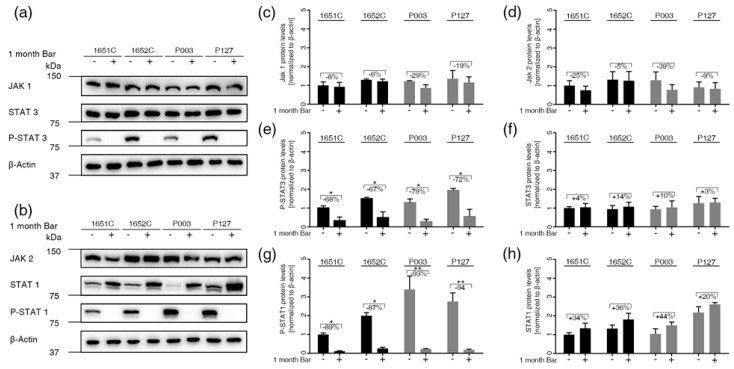
Status of the JAK-STAT signaling pathway in normal and HGPS cells treated with 1 μM Bar. (**a**,**b**) Representative images of western blots for JAK1/2, STAT1/3, P-STAT1/3 and β-actin in normal (GMO1651c, and GMO1652c) and HGPS (HGADFN003, and HGADFN127) cells treated as indicated. Cultures exhibiting <5% senescence were treated with Bar or DMSO for a period of one month. (**c**) Quantification of JAK1, (**d**) JAK2, (**e**) P-STAT3, (**f**) STAT3, (**g**) P-STAT1, and (**h**) STAT1. Graphs show the mean ± SD. Protein levels were compared by two-tailed t test (* *p* < 0.05, ** *p* < 0.01, *** *p* < 0.001, *n* > 3).

Next, we assessed the status of JAK1/2 and STAT1/3 in normal and HGPS cultures with ≤ 5% and ~15% SNS prior to treatment and after treatment with 1 μM Bar for one month (Figure 4). JAK1 and JAK2 protein levels tended to decrease in Bar-treated cells, but this change was not significant (Figure 4a–d). The levels of total STAT1 and STAT3 remained constant in one-month Bar-treated cells (Figure 4a,b,f,h). Phosphorylated-STAT1 and phosphorylated-STAT3 were increased in one-month mock-treated cells but were significantly decreased in Bar-treated normal and HGPS cells (Figure 4e,g). These findings indicate that STAT1 and STAT3 are activated during replicative senescence and that Bar treatment effectively inhibits the JAK1/2-STAT1/3 signaling axis in both normal and HGPS cells.

### 3.6. Inhibition of JAK1 and JAK2 Ameliorates Age-Related Cellular Changes in Normal and HGPS Cells

**Figure 5:**
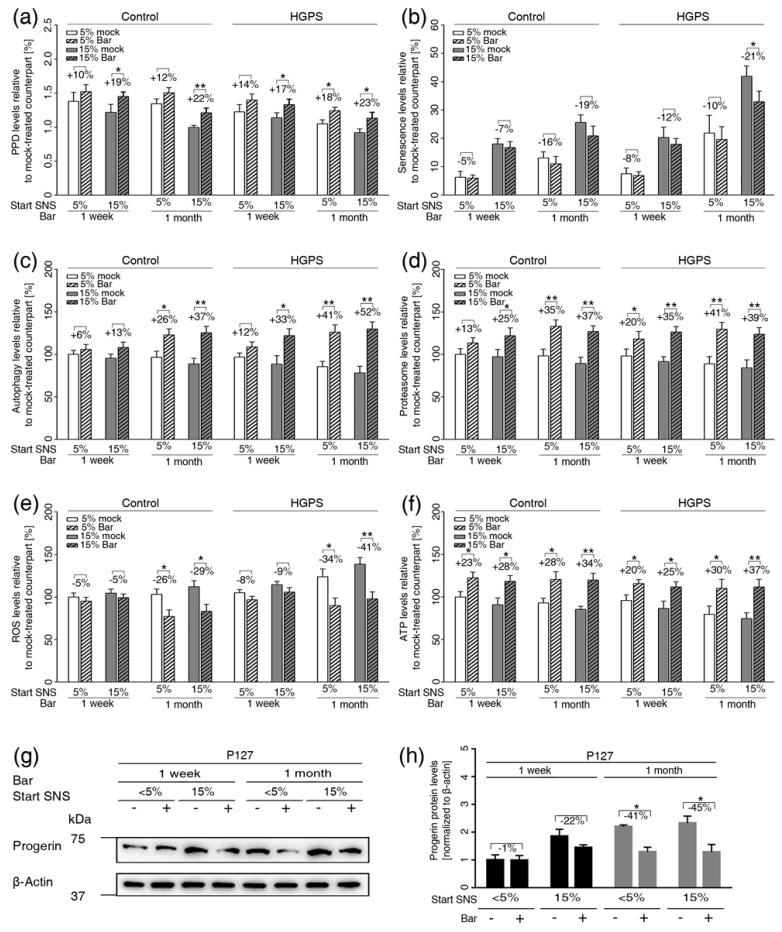
Bar treatment ameliorates normal and HGPS cellular functions during long-term treatment. (**a**) Graph shows the population doublings of control (GMO1651c, and GMO1652c) and HGPS (HGADFN003, and HGADFN127) cells. Bar (1 μM) or DMSO treatment was administrated for one week or one month as indicated. The percentage of senescence (SNS) in cultures prior to treatment is indicated. (**b**) Graph shows the percentage of SA-ß-gal-positive cells measured after treatment. (**c**) Autophagy activity was determined by measuring MDC levels using fluorescence photometry of the same cultures as in (**a**). (**d**) Proteasome activity was determined by measuring chymotrypsin-like proteasome activity using Suc-LLVY-AMC as a substrate. (**e**) Intracellular ROS levels were determined by measuring oxidized dichlorofluorescein (DCF) levels using a DCFDA cellular ROS detection assay. (**f**) Cellular ATP levels were measured using a CellTiter-Glo luminescence ATP assay. (**c**–**f**) The percent change in Bar-treated cells relative to mock-treated counterparts is indicated. (**g**) Representative images of a western blot for progerin in HGPS (HGADFN127) cells from cultures at 5% and 15% senescence that were administrated the mock or Bar treatment for one week or one month as indicated. (**h**) Quantification of the progerin signal. The percent change between Bar-treated cells and mock-treated counterparts are indicated. Graphs show the mean ± SD. Comparisons were performed by two-tailed t test (* *p* < 0.05, ** *p* < 0.01, *** *p* < 0.01, (*n* > 3)).

Normal and HGPS cultures at ≤5% and ~15% SNS were treated with 1 μM Bar or DMSO for one week or one month (Figure 5). Cultures treated with 1 μM Bar showed no presence of apoptosis (Figure S3a). Bar treatment significantly increased the growth rate of both normal and HGPS cultures at one week and one month (Figure 5a). Compared with mock treatment, one week of Bar treatment induced a minor decrease in senescence compared to mock-treated counterparts (Figure 5b). However, cultures containing ~15% SNS prior to Bar treatment for one month showed a reduction in senescence of approximately 20% (Figure 5b). Bar treatment improved the proliferative lifespan and delayed the senescence of both normal and HGPS cells after one month of treatment. 

To assess the efficacy of Bar on cellular homeostasis, we examined proteostasis, mitochondrial function, and progerin levels. The basal levels of autophagy in young (≤5% SNS) HGPS cultures were similar to those in 15% SNS control cultures (Figure 5c). The basal levels of autophagy were even lower in HGPS cultures exhibiting 15% SNS (Figure 5c). Bar treatment enhanced autophagy levels in both normal and HGPS cultures independently of their senescence index after one week of treatment, and autophagy remained increased after one month of Bar treatment (Figure 5c). Next, we assessed proteasome activity and found that its levels were similar in young (≤5% SNS) cultures of both cell types (Figure 5d). However, normal and HGPS cultures with ~15% SNS showed comparable decreases in proteasome activity (Figure 5d). Bar treatment enhanced proteasome activity in both normal and HGPS cells after one week and one month of treatment, independently of the senescence index prior to treatment (Figure 5d). 

In accordance with previous reports [15], we also found that reactive oxygen species (ROS) levels were increased in HGPS cultures harboring ~15% SNS (Figure 5e). Bar treatment effectively reduced ROS levels in both cell types treated for one month (Figure 5e). Moreover, we found that ATP levels were reduced in both cell types exhibiting ~15% SNS prior to treatment (Figure 5f). Bar treatment similarly increased intracellular ATP levels in both cell types, one week and one month after treatment (Figure 5f).

Because progerin is degraded through autophagy [12,15], we determined progerin levels in Bar-treated cultures (Figure 5g,h). After one month, Bar treatment effectively reduced progerin levels by approximately 40% in HGPS-treated cells (Figure 5g,h). Then, we investigated the impact of Bar treatment on HGPS nuclear morphology (Figure S3b,c). HGPS nuclei are highly dysmorphic and exhibit numerous alterations, including nuclear envelope abnormalities [15]. Twenty days of Bar treatment significantly reduced the number of dysmorphic nuclei in both normal and HGPS cultures (Figure S3b). Additionally, in mock-treated cultures, numerous HGPS nuclei exhibited increased progerin accumulation at the nuclear envelope, with aggregates in certain areas as revealed when stained with anti-progerin antibodies (Figure S3c). Overall, the number of bright progerin-positive nuclei and the signal intensity of these nuclei were reduced in Bar-treated HGPS cells compared with mock-treated HGPS cells. Collectively, Bar treatment ameliorates HGPS nuclear morphology by reducing progerin levels. Taken together, these results demonstrate that Bar inhibition of the JAK-STAT signaling pathway restored proliferation, proteostasis and mitochondrial function, which are known hallmarks of cellular aging. 

### 3.7. JAK-STAT Inhibition Reduces Proinflammatory Factors

Given the role of the JAK/STAT pathway in inflammation, we examined whether inhibiting the JAK/STAT pathway rebalanced the gene expression of the 17 genes identified by text mining. We treated young (≤5% SNS) normal and HGPS cultures with 1 μM Bar or vehicle for one month and determined the percentage of senescence as indicated (Figure S4). Bar treatment reduced the number of SNS and prevented the upregulation of most of cytokines/ chemokines known as SASPs (CCL2/MPC1, CXCL8/IL8, IFNG, IL4, IL6, IL18, LEP, TNFα), with the most significant reductions in IL6, CXCL8/IL8, IL18, and CCL2/MPC1, which are important proinflammatory factors (Figure S4). Hence, TGFB1, TRAF1, ICAM1, FAS, and CRP were also decreased in Bar-treated cells (Figure S3). The levels of IGF1 and PPARG were increased in Bar-treated cells and therefore ameliorated in both cell types (Figure S4i,n). Collectively, these results indicate that Bar treatment decreasing the expression of several SASPs, which might contribute to the delayed senescence in both normal and HGPS cells. 

### 3.8. Etoposide-Induced DNA Damage Overactivates the JAK-STAT Pathway in both Normal and HGPS Cells

**Figure 6:**
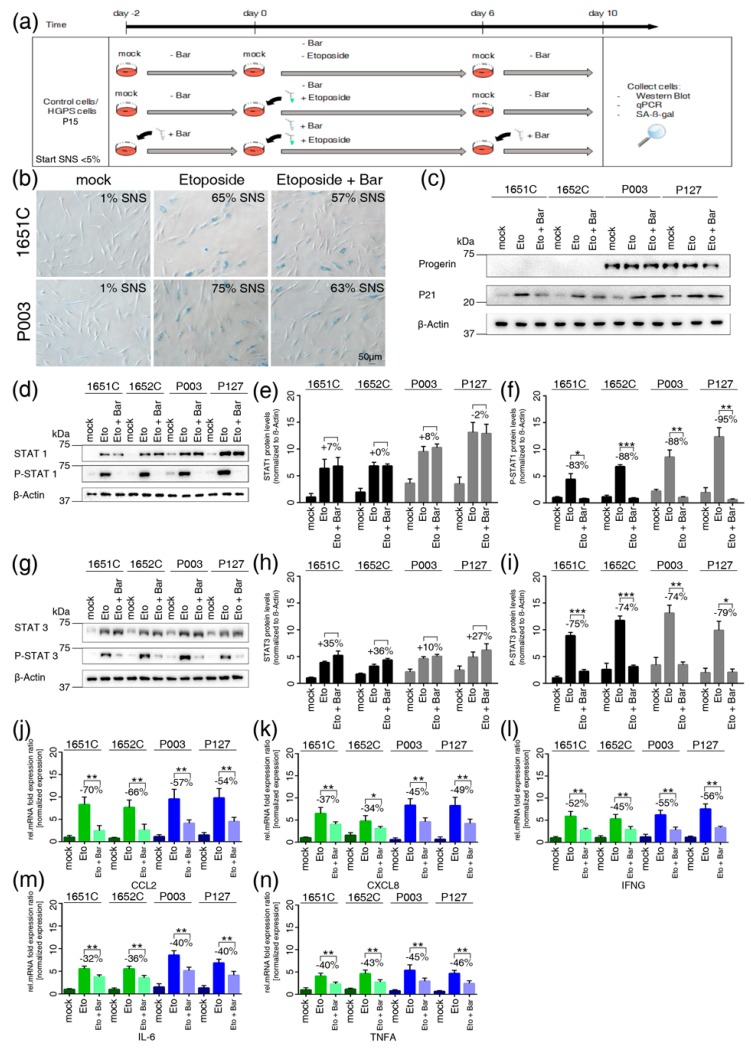
Etoposide treatment activates the JAK-STAT signaling pathway in HGPS and normal cells. (**a**) Schematic representation of the etoposide treatment protocol. All treatments started with cultures exhibiting <5% senescence (SNS). Cells were either pretreated with or without 1 µM Bar for two days and then expose to medium with or without etoposide in the presence or absence of Bar for a period of six days. Next, cells were grown for four days with or without Bar as indicated. (**b**) Representative images of SA-ß-gal-positive cells in mock-, etoposide- and etoposide + Bar-treated cultures (GMO1651c and HGADFN003) are shown. The percentages of senescence (SNS) after treatment are indicated. (**c**) Representative images of western blots for progerin, p21 and ß-actin in normal (GMO1651c, and GMO1652c) and HGPS (HGADFN003, and HGADFN127) cells treated as indicated. (**d**) and (**g**) Representative images of western blots for JAK1/2, STAT1/3, P-STAT1/3 and ß-actin in normal (GMO1651c, and GMO1652c) and HGPS (HGADFN003, and HGADFN127) cells treated as indicated. (**e**) Quantification of STAT1, (**f**) p-STAT1, (**h**) STAT3, and (**i**) p-STAT3. The percent change between etoposide- and etoposide+Bar-treated cells is indicated. (**j**–**n**) Quantitative real-time PCR analysis of CCL2, CXCL8, IFNG, IL6, and TNFα in cells treated as indicated. Relative expression was normalized to the expression of GAPDH. Graphs show the mean ± SD. Comparisons were done by two-tailed t test (* *p* < 0.05, ** *p* < 0.01, *** *p* < 0.001). All experiments were repeated at least three times (*n* > 3).

To further investigate the role of JAK-STAT signaling in the development of cell senescence excluding replicative senescence, we exposed young (≤5% SNS) cultures to etoposide, an inhibitor of topoisomerase that causes significant DNA damage and cellular senescence [35]. Normal and HGPS cells were treated with 5 μM etoposide for six days and then switched to normal media for four days (Figure 6a). SA-β-Gal staining indicated that approximately 65% of the control cells and 75% of the HGPS cells were senescent on day 10 of etoposide treatment (Figure 6b). In accordance with the increased senescent population, p21 levels were increased in etoposide-treated cells (Figure 6c). In cultures pretreated with Bar and cotreated with etoposide, the percentages of SNS were decreased by approximately 8% in normal and 12% in HGPS cultures (Figure 6b). During the treatment period, progerin levels remained unchanged in HGPS cells (Figure 6c). 

Then, we assessed the status of STAT1 and STAT3. The basal levels of total STAT1 and STAT3, including their phosphorylated forms, were low in mock-treated cultures (Figure 6d–i). However, after etoposide treatment, a sharp increase in total STAT1 and, to a lesser extent STAT3 was detected in both normal and HGPS cells (Figure 6e,h). Hence, etoposide treatment also induced high levels of phosphorylated STAT1 and phosphorylated STAT3 indicating that these proteins were activated (Figure 6f–i). In contrast, combined treatment with Bar and etoposide effectively blocked the phosphorylation of STAT1 and STAT3 (Figure 6f–i). Collectively, these findings demonstrate that high levels of DNA damage induce the overactivation of JAK1/2-STAT1/3 signaling, as observed during replicative senescence. In addition, gene expression profiling of CCL2/MPC1, CXCL8/IL8, IFNG, IL6 and TNFα showed that these genes were upregulated in etoposide-treated cells compared to mock-treated cells, in accordance with the increased senescence index of the cultures (Figure 6j–n). In contrast, the levels of these proinflammatory factors were decreased in cells cotreated with Bar (Figure 6j–n). Taken together, these findings indicate that similar to replicative senescence, DNA damage-inducing agents mediate senescence through the overactivation of JAK-STAT signaling, and Bar treatment delays senescence and decreases the expression of proinflammatory SASPs.

## 4. Discussion

In this study, we used a text mining approach to identify the molecular signatures underlying four distinct age-related pathologies (vascular disease, arthritis, alopecia and lipodystrophy) that affect elders and HGPS patients to identify potential converging and overlapping signaling pathways. We found that proinflammatory cytokine-mediated activation of the JAK1/2-STAT1/3 pathway was a critical effector in the pathogenesis of these four conditions. Hence, we identified an inflammatory signature of 17 factors, 14 of which are targets of the STAT1 and STAT3 transcription factors. These 14 inflammatory markers belong to SASP, and are therefore secreted by SNS cells [36]. Collectively, our findings indicate a critical role of chronic inflammation, senescence and JAK-STAT signaling overactivation in the development of these four age-related conditions and HGPS. 

Our findings are in accordance with emerging evidence indicating that chronic inflammation develops with age [37]. An inflammatory signature was detected in blood samples from individuals suffering from diverse age-related pathologies, including HGPS patients [38]. The 17 genes identified herein could serve as biomarkers for scoring the stage and severity of these four conditions and HGPS. 

Inflammation is normally induced during infection or tissue injury and can be tracked by increased levels of proinflammatory factors such as cytokines (IL6, IL8, IL18, and TNFa) in plasma samples. When inflammation persists, it can become destructive to tissues and organs [39]. If the cytokines balance is altered, then chronic inflammation (“inflamm-aging”) is instated and leads to aging and the development of age-related diseases [40]. A potential cause of chronic inflammation is the accumulation of SNS in particular tissues and organs [32]. Hence, senescence can develop in different cell types, such as immune cells [41], stem cells [42], smooth muscle cells, endothelial cells, and fibroblasts in atherosclerotic plaques [43], adipocytes in fat tissue [44], keratinocytes and fibroblasts in elderly skin [31], and synovial fibroblasts in arthritic joints [45]. Therefore, the tissues in which SNS accumulates determines which age-related pathology will develop, and the accumulation of SNS over time will worsen the condition. 

Studies have shown that inflammation is first activated by the NF-κB pathway, inducing the secretion of IL6, IL-1α, TNFα and other cytokines that can further propagate intracellular proinflammatory signaling cascades [28]. In this context, our study revealed the overactivation of JAK-STAT signaling in SNS using an ex vivo cell-based aging model. We used primary fibroblast cultures on the basis that all four conditions affect mesenchymal-derived tissues, including the heart, vascular vessels, skin, bone and joints, which all contain resident immune cells, tissue-specific cell types and fibroblast subtypes. While fibroblasts are highly heterogeneous, they all share a similar property; they express numerous factors and participate in different functions, such as wound healing, repair, remodeling, fibrosis and the maintenance of stem cells [32]. Because fibroblasts are present in all the tissues related to the four investigated diseases, their increased senescence might play a key role in the development of these pathologies.

In this study, we used long-term primary fibroblast cultures to examine the properties of normal and HGPS fibroblasts by comparing cultures with a similar senescence index instead of simply taking into account the passage number or the population doublings. Because HGPS cells prematurely enter senescence due to the expression of progerin, the senescence score was critical to revealing several important findings. Indeed, cultures with less than 5% senescence from both HGPS and normal individuals exhibited similar growth rates, with minor differences in autophagy levels, proteasome activity, and ROS and ATP levels. In both normal and HGPS cultures with 15% senescence or higher, all parameters mentioned above were altered. These results indicate that concomitantly increased SNS, alterations in proteostasis and mitochondria function occur in both normal and HGPS cultures. These functional changes are associated with senescence and are not specifically associated with progerin expression in HGPS cells. This difference finding is due to the lack of scoring senescence cell levels in tested cultures in previous studies. We discovered this parameter because comparative studies between normal and HGPS cultures with matching passage numbers showed inconsistent levels of p-STAT1 and p-STAT3 even within the same cell strain. We identified the striking similarities of senescing HGPS and normal cells because we rigorously scored the rate of senescence of the cultures. This strategy showed that normal and HGPS cultures at analogous senescence indexes exhibited similar mRNA levels of genes encoding proinflammatory factors (CRP, CCL2, IL8, C3, TRAF1, IL18, IL6, and TNFα), indicating that these SASPs were similarly expressed during normal and premature aging (HGPS). Hence, the levels of phosphorylated forms of STAT1 and STAT3 were similarly increased during senescence, indicating that in both cell types, the JAK1/2-STAT1/3 signaling axis was activated during replicative senescence. Because senescence can occur in response to various stresses other than telomere attrition, the senescence of HGPS and normal fibroblast cultures was induced by DNA damage-induced senescence using etoposide treatment. Remarkably, the JAK1/2-STAT1/3 signaling pathway was also overactivated in the etoposide-treated cultures. In both the normal and HGPS cells treated with etoposide, the levels of phosphorylated STAT1 and STAT3 increased abruptly in SNS. Thus, the activation of the JAK1/2-STAT1/3 signaling axis elicited the upregulation of a targeted set of inflammatory cytokines (IL6, IL4, IL8, IL18, TNFα, CCL2, IFNy and C3), including the adhesion molecules ICAM-1, and CRP, as observed during replicative senescence. Therefore, the overactivation of the JAK-STAT pathway in SNS appears to be a general mechanism of senescence that sustains the production of proinflammatory factors independently of the stress that induces cellular senescence. 

To target the pro-inflammatory phenotype, we tested the effect of Bar, a specific inhibitor of JAK1 and JAK2 [17]. Bar treatment efficiently prevented the activation of the JAK1/2-STAT1/3 signaling axis during replicative senescence and in cells exposed to a DNA-damaging agent (etoposide). One month of Bar treatment improved growth rate, delayed senescence, ameliorated proteostasis and mitochondrial function, significantly decreased proinflammatory marker levels and reduced progerin levels in HGPS cells. Consequently, Bar treatment was able to rebalance the homeostasis of normal and HGPS cells, correcting several hallmarks of aging.

## 5. Conclusions

The text mining approach allowed us to identify chronic inflammation and JAK-STAT signaling overactivation as common etiologies in the pathogenesis of vascular disease, arthritis, lipodystrophy, and alopecia, which affect HGPS patients. Using an ex vivo cell-based aging model, we provide evidence that the inhibition of JAK1/2-STAT1/3 signaling could ameliorate chronic inflammation and delay senescence. Our findings support the use of Bar as a valuable therapeutic strategy for children with HGPS and for individuals suffering from these four age-related conditions and possibly other age-related pathologies.

## Figures and Tables

**Table 1:** Transcription factors that regulate the majority of the 17 genes identified by text mining. Each of the 17 genes was searched with the TRRUST version 2 (https://www.grnpedia.org/trrust/), TfactS (http://www.tfacts.org/) and Regulatory Circuits database (http://regulatorycircuits.org/). A total of 140 transcription factors were identified and each transcription factor was associated with at least one of the 17 genes (Supplementary Table S3). The transcription factors linked to most of the 17 genes as determined by direct evidence are shown.

Gene	NF-κB1	Rela	STAT3	STAT1	STAT5A	STAT5B	STAT6
C3							
CCL2	√	√	√	√			
CRP	√	√	√				
CXCL8	√	√	√	√			√
FAS	√	√	√	√			
HMOX1	√	√	√	√			
ICAM1	√	√	√	√			
IGF1			√		√	√	
IL6	√	√	√	√	√		
IL18	√	√					
PPARG	√			√		√	
TGFB1	√		√				
TNF	√	√	√		√	√	
TRAF1	√	√					
LEP			√				
IL4	√	√					√
IFN-G	√	√	√	√	√	√	

## Supplementary Materials

The following are available online at https://www.mdpi.com/2073-4409/8/10/1276/s1, Figure S1: Real-time PCR analysis of the JAK and STAT gene families in normal and HGPS cells, Figure S2: Cytotoxicity and stability of Bar in a cell-based aging model, Figure S3: Bar treatment ameliorates HGPS nuclear morphology, Figure S4: Real-time PCR analysis of the 17 genes identified by text mining in normal and HGPS cells treated with Bar for 1 month, Table S1: Dataset File 1: Excel file of the list of genes associated with vascular disease, arthritis, lipodystrophy and alopecia derived from the text mining analysis, Table S2: Differential regulation of the 17 genes altered in all four conditions, Table S3: Dataset File 2: Excel file of the identified list of transcription factors that regulate the 17 genes derived from text mining, Table S4: Primers used for the real-time quantitative PCR analysis of the 17 genes identified by text mining.
